# Maintenance of Immune Homeostasis through ILC/T Cell Interactions

**DOI:** 10.3389/fimmu.2015.00416

**Published:** 2015-08-13

**Authors:** Nicole von Burg, Gleb Turchinovich, Daniela Finke

**Affiliations:** ^1^Department of Biomedicine, University of Basel, Basel, Switzerland; ^2^University of Basel Children’s Hospital, Basel, Switzerland

**Keywords:** innate lymphoid cell, cytokine, T helper cell, immune response, antigen presentation

## Abstract

Innate lymphoid cells (ILCs) have emerged as a new family of immune cells with crucial functions in innate and adaptive immunity. ILC subsets mirror the cytokine and transcriptional profile of CD4^+^ T helper (T_H_) cell subsets. Hence, group 1 (ILC1), group 2 (ILC2), and group 3 (ILC3) ILCs can be distinguished by the production of T_H_1, T_H_2, and T_H_17-type cytokines, respectively. Cytokine release by ILCs not only shapes early innate immunity but can also orchestrate T_H_ immune responses to microbial or allergen exposure. Recent studies have identified an unexpected effector function of ILCs as antigen presenting cells. Both ILC2s and ILC3s are able to process and present foreign antigens (Ags) via major histocompatibility complex class II, and to induce cognate CD4^+^ T cell responses. In addition, Ag-stimulated T cells promote ILC activation and effector functions indicating a reciprocal interaction between the adaptive and innate immune system. A fundamental puzzle in ILC function is how ILC/T cell interactions promote host protection and prevent autoimmune diseases. Furthermore, the way in which microenvironmental and inflammatory signals determine the outcome of ILC/T cell immune responses in various tissues is not yet understood. This review focuses on recent advances in understanding the mechanisms that coordinate the collaboration between ILCs and T cells under homeostatic and inflammatory conditions. We also discuss the potential roles of T cells and other immune cells to regulate ILC functions and to maintain homeostasis in mucosal tissues.

## Introduction

Adaptive immune responses are tightly controlled by the selection of the T and B cell receptor repertoire and by transcriptional networks regulating commitment, expansion, and contraction of the responses. Upon cognate antigen (Ag)–peptide–major histocompatibility complex (MHC) recognition Ag-specific T helper (T_H_) cells proliferate and differentiate into effector T_H_ cell subsets with distinguishable cytokine profiles. Almost 30 years ago, interferon (IFN)-γ-secreting T_H_1 cells were discriminated from T_H_2 cells, whose cytokine profile includes interleukin (IL)-4, IL-5, and IL-13 (1). Additional subsets of T_H_ cells, such as T_H_17 (2), regulatory T (T_reg_) cells (3), T_H_9 (4), T follicular helper cells (5), and more recently granulocyte-macrophage colony-stimulating factor (GM-CSF) producing T_H_ cells (6–8), were described.

In the past 5 years, new subsets of innate immune cells have emerged as a first line of defense at mucosal barriers. Like conventional natural killer (cNK) cells, they belong to the lymphoid lineage and develop from common lymphoid progenitor (CLP) cells but unlike T and B cells, they lack rearranged Ag-receptors. Hence, they were termed innate lymphoid cells (ILCs). ILCs are found in various tissues including mucosa, lymphoid tissue, liver, skin, and fat. They depend on the expression of the common cytokine receptor γ chain (γ_c_ chain) and the transcriptional repressor inhibitor of DNA binding 2 (ID2) for their development (9–11). The factors involved in regulating different stages of ILC commitment from CLPs have been recently reviewed in Ref. (12). ILCs resemble T_H_ cells in their developmental requirements, transcriptional regulation, and in their cytokine secretion pattern. Thus, they were classified into three groups, which are able to immediately react to microbial and inflammatory challenge with cytokine production thereby limiting pathogen spread and tissue injury (9). Group 1 ILCs consist of cNK cells and so-called helper ILC1s; both secrete the T_H_1-type cytokine IFN-γ. Group 2 ILCs are characterized by the production of T_H_2-type cytokines IL-4, IL-5, and/or IL-13. Group 3 ILCs include fetal lymphoid tissue-inducer (LTi) cells, as well as adult ILC3s either expressing the natural cytotoxicity receptor (NCR) NKp46 (NCR^+^ILC3s) or lacking this molecule (NCR^−^ILC3s). Cells within this group produce the T_H_17-type cytokines, IL-17 and/or IL-22 (9). The classification into ILC1, 2, and 3 is sometimes unhelpfully restrictive because ILCs have the potential to modulate their phenotypic and transcriptional signature upon activation and inflammation. When exposed to inflammatory conditions, NCR^−^ILC3s can produce IFNγ (13, 14), and NCR^+^ILC3s are able to convert into IFNγ-producing ILC1-like cells (15, 16). Moreover, in multiple sclerosis patients, blockade of CD25 (IL-2Rα) induces phenotypic changes of ILC3s toward cNK cells (17). Additional evidence for heterogeneity among ILC subsets comes from clonal analysis in humans demonstrating that the spectrum of cytokines produced by ILC3s is diverse (18) and in some cases, both ILC2 and ILC3 cytokines are produced (19). Finally, environmental factors, such as retinoic acid, short chain fatty acids, vitamins, aryl hydrocarbon receptor (AHR) ligands, stearyl sulfate, and probably bacterial metabolites, can shape ILC phenotypes and functions (20–24). Together, these data now provide convincing evidence that, similar to T_H_ cells, ILCs have a degree of plasticity in their cytokine profile. As for T_H_ cell commitment, cytokine-mediated conditioning, as well as epigenetic (25, 26) and transcriptional regulation (27) may account for changes of ILC subset-determining transcription factors and cytokines.

The biological relevance of ILCs is based on their capacity to sense environmental and inflammatory signals, and to respond with the secretion of cytokines important for immune defense, allergic reactions, and tissue repair. Recent data provide additional evidence that ILCs can condition T cell responses, either through cytokines, direct cell–cell contact, or through effects on accessory cells. This review will focus on the effects of ILC–T cell interactions for maintaining immune homeostasis. We will highlight major questions on how ILCs may cooperate with T cells thereby regulating T cell responses.

## Induction and Skewing of T Cell Responses

Dendritic cells (DCs) are professional Ag-presenting cells (APCs) known for their robust capacity to activate naïve T cells and to modulate innate and adaptive immune responses (28). Distinct DC subsets have decisive roles in engaging pathways responsible for skewing the type of effector T_H_ cell response (29, 30). Moreover, DCs can suppress immune responses in order to maintain peripheral immune homeostasis and tolerance to self-Ags (31). As a key step in shaping the type of T_H_ cell response, cytokines secreted by innate immune cells including APCs can account for the expression of T_H_ subset-specific transcription factors (32). For example, IL-12 activates signal transducer and activator of transcription (STAT)-4 and induces the expression of the T-box transcription factor T-bet, which is critical for T_H_1 cell commitment (33, 34). T-bet expression and T_H_1 cell differentiation are further promoted by IL-2 (35). IL-4 induces STAT6 activation, which enhances Gata3 expression thereby initiating differentiation into T_H_2 cell lineage (36). Additionally, IL-2 signaling followed by STAT5 activation plays a crucial role in T_H_2 cell commitment by the induction of IL-4 transcription (37, 38). IL-6 signaling through STAT3, together with transforming growth factor (TGF)-β, induces retinoic acid-related orphan receptor (ROR)-γt expression and consequently the differentiation of pathogenic T_H_17 cells from naïve T_H_ cells (39). A key issue in establishing immune homeostasis is the induction of T_reg_ cells that prevent immunopathology by maintaining tolerance. In addition, active suppression of inappropriate T cell responses is mediated by the induction of immune-regulatory cytokines, such as IL-10 (40), the expression of inhibitory receptors including cytotoxic T-lymphocyte-associated protein (CTLA)-4 or programed cell death (PD)-1 or the lack of co-stimulation and bystander signals. Altogether, cytokines and activating or inhibiting receptors of innate immune cells are pivotal for generating and conditioning T_H_ cell responses.

## Group 1 ILCs

The group 1 ILCs comprised cNK cells and helper ILC1s. Both subsets secrete IFNγ and express the transcription factor T-bet (15, 16, 41–43). The expression of Eomesodermin (Eomes) is considered as a key factor for distinguishing cNK cells (Eomes^+^) from ILC1s (Eomes^−^) (43). However, splenic NK1.1^+^ CD127 (IL-7Rα)^+^ cells, which are in some studies referred to as ILC1s, express considerable levels of Eomes (44). Nfil3, another transcription factor, has been attributed a role in specifying cNK cells versus ILC1s. Although important for the development of all ILC lineages, studies of Nfil3-deficient mice (42, 45, 46) revealed that cNK cells have greater dependency on Nfil3 than ILC1s (47, 48). This is probably due to direct transcriptional control of Eomes expression by Nfil3 (49). Thus, NK cells resident in the salivary gland appear to be a prototype of ILC1s, as they also do not require Nfil3 for their development (48). Cells defined as ILC1s in the intestinal epithelium in humans and mice express the epithelial homing marker CD103 and readily produce IFNγ upon stimulation (41). CD103^+^ intraepithelial ILC1s, similar to cNK cells, express Eomes and T-bet, and are Nfil3-dependent, but in contrast to cNK cells do not require IL-15 for their development. Phenotypically, cNK cells express DX5 and, unlike most ILC1s, lack Trail or CD127 expression (43, 47, 48). Some ILC1-like cells derive from RORγt^+^ ILC3s by a process that is accompanied by the loss of RORγt expression and the upregulation of T-bet in both mice and humans (15, 16, 50). Future research on T-bet^+^ IFNγ-secreting subsets will help to clarify the developmental and functional relationship of group 1 ILCs.

## Group 1 ILC–T Cell Interactions

Unlike group 2 and group 3 ILCs, murine cNK cells and ILC1s do not express MHC class II (MHC II) molecules, thus being incapable of direct Ag-dependent interaction with CD4^+^ T_H_ cells (Table 1). Nevertheless, in recent years, a number of reports described new aspects of a direct crosstalk between T and cNK/ILC1 cells. Several studies defined a regulatory role for cNK cells in controlling T cell-dependent immune responses by direct cytotoxic activity toward CD4^+^ and CD8^+^ T cells (51–53), as well as toward APCs required for T cell priming. Two recent publications demonstrated that type 1 IFN confer the resistance to cNK cell-mediated lysis of activated CD8^+^ T cells (54, 55). CD8^+^ T cells isolated from IFN-α-receptor-1-deficient (*Ifnar1*^−^*^/^*^−^) mice were preferentially targeted by cNK cells resulting in the elimination of cytotoxic CD8^+^ T cells in response to viral infection through a perforin-dependent pathway. Another study proposed a role for NKp46 in limiting graft versus host disease (GVHD) (56), although it has remained obscure whether NKp46 is required for the direct killing of host-reactive T cells, or if it operates via targeting of accessory APCs. More recently, Schuster et al. reported that cNK cells specifically limit the number of virus-reactive CD4^+^ T cells in a model of chronic murine cytomegalovirus (MCMV) infection in the salivary gland (57). Intriguingly, this process is dependent on the TNF-superfamily ligand Trail, which is, in addition to NKp46 also expressed by ILC1s. This suggests a possible contribution of ILC1s to the processes described above. Additionally, in humans, activated cNK cells could be shown to positively regulate CD4^+^ T_H_ cell activity (58). cNK cells stimulated by cytokines or through activating receptors were shown to upregulate the co-stimulatory molecules, OX40L and members of B7 family (CD80/CD86). Interaction with such cNK cells led to augmented IFNγ production and enhanced T cell receptor-dependent proliferation of autologous CD4^+^ T_H_ cells.

**Table 1:** **Phenotype of mouse and human ILCs**.

	Mouse				Human			
	cNK	ILC1	ILC2	ILC3	cNK	ILC1	ILC2	ILC3
**SURFACE MOLECULES**								
CD90	+	+	+	+	ND	ND	ND	ND
CD127	−^a^	+	+	+	lo	−^a^	+	+
CD117	lo	+	+^c^	+	lo	sub^l^	±	+
NK1.1	+	+	−	lo	+	+	+	+^o^
NKp46/NKp44	+	+	+	sub	sub	+^a^	−	sub^o^
CD25	−	−^b^	+	+	+	−	+	+^o^
ST-2	−	−	+^d^	−	−	−	+	−
Sca-1	−	−	+^e^	lo	ND	ND	ND	ND
**TRANSCRIPTION FACTORS**								
ID2	+	+	+	+	ND	ND	ND	+^o^
Gata3	−	lo	+	lo	lo	lo	+	lo
RORγt	−	−	lo	+	−	lo	lo	+
T-bet	+	+	−	sub	+	+	−	−
Eomes	+	−	−	−	+	−	−	−
NFIL3	+	+	+	+	ND	ND	ND	ND
**MOLECULES INVOLVED IN ILC–T CELL INTERACTION/ILC ACTIVATION**								
CD69	lo	lo	−^f^	ind, +^h^	+	sub^m^	sub^m^	sub^m, p^
MHC class II	−	−	+	+^i^	ind, +^k^	ND	+^n^	+
CD80	−	−	ind^g^	ind^j^	ind, +^k^	ND	+^n^	ND
CD86	−	−	ind^g^	ind^j^	ind, +^k^	ND	+^n^	ND
CD40	−	−	−	ind^j^	−	ND	ND	ND
CD30L	−	lo	−	+	ind, +^k^	ND	ND	ND
OX40L	−	−	−	+	ind, +^k^	ND	ND	+
ICOS	−	−	+	ND	ind, +^k^	ND	+	+
ICOSL	−	lo	+	ND	ND	ND	+	lo
RANKL	−	−	ND	+	−	ND	ND	+
TRAIL	−	+	ND	lo	ind, +^k^	ND	ND	ND

*+ indicates expression; − indicates no expression; lo indicates low expression; sub indicates expression on a subset; ind indicates activation-induced expression; ND indicates expression is not determined*.

*^a^Expressed in certain tissues; ^b^Intestinal ILC1s are CD25^+^ (44); ^c^Skin ILC2s are CD117^−^ (62); ^d^Small intestinal ILC2s are ST-2^−^ (73); ^e^Liver ILC2s are Sca-1^−^ (71); ^f^Fat-associated lymphoid cluster-derived and intestinal ILC2s are CD69^+^ (69); ^g^Expressed on mediastinal LN-derived ILC2s from IL-33 treated mice (93); ^h^Expressed on splenic ILC3s under inflammatory conditions (130); constitutively expressed on intestinal ILC3s (44); ^i^Expression increased on splenic ILC3s under inflammatory conditions (130); ^j^Expressed on splenic, but not intestinal ILC3s under inflammatory conditions (130, 133); ^k^Expressed after activation (159, 160); expressed at steady state (161); ^l^Molecule expressed on certain subsets (16); ^m^Human peripheral blood ILCs heterogeneously express CD69 (162); ^n^Human ILC2s express CD80/CD86 and HLA-DR (93); ^o^Human ILC population resembling ILC3s (122); ^p^Human splenic ILCs are CD69^+^ (122)*.

Conventional natural killer/ILC1 and T cell crosstalk operates in a reverse direction as well. Two studies showed that T_reg_ cells play an important role in keeping cNK cell activity in check (59, 60). Gasteiger et al. demonstrated that upon depletion of T_reg_ cells, cNK cells become hyper-responsive toward MHC I-deficient target cells that are recognized via missing-self mechanism. This was attributed to the increased availability of IL-2 produced by activated CD4^+^ T cells (59). Another report demonstrated in a genetic model of type 1 diabetes that the acute removal of T_reg_ cells leads to the accumulation of activated cNK cells in pancreatic islets (60). On the contrary, in this experimental setting, depletion of T_reg_ cells did not result in an increase of IL-2 secretion by CD4^+^ T_H_ cells, but more likely increased the availability of IL-2 to cNK cells by decreasing IL-2 consumption by T_reg_ cells. Interestingly, the accumulating cNK cells express CD127 (61) and might therefore constitute an “ILC1-like” subset. These studies provide the first example of T_reg_ cell-dependent control of cNK cell and possibly ILC1 activity. Given the importance of IL-2 for the expansion of other ILC subsets (45, 62), T_reg_ cells might also be involved in controlling their activity. Taken together, these findings illustrate the reciprocal immuno-regulatory relationship between group 1 ILCs and T cells.

## Group 2 ILCs

ILC2s are the most homogenous ILC subset albeit with a specific phenotypic signature in the lung and intestine (44, 63). They express CD127, CD90.2 (Thy1), various levels of CD25, and the IL-33-receptor subunit ST2 (Table 1). The development of ILC2s depends on the transcription factors, ROR-α, Gata3, and T cell factor (TCF)-1 (64–67). ILC2s in both humans and mice secrete T_H_2-type cytokines IL-4, IL-5, and/or IL-13 in response to IL-9, IL-25, IL-33, and thymic stromal lymphopoietin (TSLP), as well as during pulmonary inflammation or infection with *Nippostrongylus brasiliensis*, a helminth controlled by T_H_2-type cytokine responses (63, 68–78). In addition to ILC2s, another cell type, the multipotent progenitor type 2 (MPP^type2^) is described. MPP^type2^ cells exhibit similar phenotypic and functional characteristics with ILC2s (79), but do not produce T_H_2-type cytokines in response to IL-33 (80). The release of T_H_2-type cytokines by ILC2s is not only involved in *N. brasiliensis* expulsion (81) but can also trigger airway inflammation and allergic responses in humans (82–84). Together, ILC2s share developmental and inducible cytokine signatures with T_H_2 cells suggesting a role in type 2 immune responses.

## Group 2 ILC–T Cell Interactions

Type 2 immune responses are severely impaired in IL-4-receptor-α-deficient (*Il4Rα*^−^*^/^*^−^) and IL-4-deficient (*Il4*^−^*^/^*^−^) mice indicating that IL-4 has a role in T_H_2 cell differentiation (85, 86). Further, the accumulation of T_H_2 cells after *N. brasiliensis*/ovalbumin challenge is dramatically reduced in IL-4 and IL-13-double-deficient (*Il4*^−^*^/^*^−^*Il13*^−^*^/^*^−^) mice as compared to wild type (*WT*) mice (87). T_H_2 cell differentiation is most likely initiated by innate immune cells, which become activated in the early phase of immune responses. Beside basophils and mast cells (88–90), it is now well established that ILC2s can secrete IL-4 suggesting a role for these cells in the induction of T_H_2 cell differentiation and type 2 immune responses. Indeed, several reports provide evidence that ILC2s and CD4^+^ T cells cooperate at multiple levels (91–97). In mice, which either have dramatically reduced numbers or a complete lack of ILC2s, the generation of type 2 immune responses upon *N. brasiliensis* infection, challenge with house dust mite Ag or with protease-allergen papain is impaired indicating a contribution of ILC2s to T_H_2 cell responses (91, 93, 95). The addition of ILC2s to cultures of naïve CD4^+^ T cells promotes the differentiation into T_H_2 cells, while inhibiting the differentiation into T_H_1 cells even in the presence of IL-12, a cytokine that drives T_H_1 differentiation (33, 34, 92). In line with this finding, type 2 cytokines are not detectable when T_H_ cells are co-cultured with ILC2s unable to secrete IL-4 (94). On the other hand, *in vivo* differentiation of T_H_1/T_H_17 cells occurs independently of ILC2s, since mice, which lack ILC2s, show normal responses when exposed to *Saccharopolyspora rectivirgula*, a bacterium inducing T_H_1/T_H_17 inflammatory responses (95). Together, there is evidence that ILC2-derived IL-4 contributes to type 2 cytokine production of T_H_ cells, although an IL-4-independent pathway for ILC2-driven type 2 immune responses may also occur (91). Beside the direct effect of ILC2s on T_H_2 differentiation, T_H_2-type cytokines secreted by ILC2s can also affect CD4^+^ T cells indirectly via DCs. Evidence for this comes from the finding that ILC2-derived IL-13 promotes migration of DCs into lung-draining lymph nodes (LNs), where activated DCs induce the differentiation of CD4^+^ T cells into T_H_2 cells (91).

Interleukin-33, a pro-inflammatory cytokine expressed by a variety of cell types can trigger the generation of inducible regulatory T (iT_reg_) cells (98) and the activation of ILC2s to produce type 2 cytokines and amphiregulin (AREG). AREG is an epithelial growth factor that promotes restoration of airway epithelial integrity following influenza virus-induced damage (63). Importantly, analysis of ILC2-depleted, influenza virus-infected mice revealed a strong reduction in AREG mRNA suggesting that ILC2s are the main source of AREG under such inflammatory conditions. In other inflammatory models, mast cells were thought to be the major source of AREG and importantly, in these models, AREG was found to be critical for efficient T_reg_ cell function (99). In view of their abundance in the skin, lung, and colon, their strong responsiveness to IL-33, and early inflammatory signals, AREG-secreting ILC2s may have a function in tissue repair and likely also in triggering T_reg_ cell responses.

Another mechanism through which ILC2s have an influence on CD4^+^ T_H_ cells is by their ability to serve as APCs. Co-stimulatory signals via OX40 are crucial for effector/memory T cell responses and for initiating T_H_2 differentiation (100, 101). OX40-ligand (OX40L) is detectable on ILC2s, and the production of T_H_2-type cytokines in ILC2-T cell co-cultures is significantly inhibited when anti-OX40L antibodies (Abs) are added, suggesting that ILC2s promote T_H_2-responses via OX40/OX40L interactions (94). Further evidence for cell–cell interactions between ILC2s and CD4^+^ T cells is provided by the finding that human and mouse ILC2s express both inducible T cell co-stimulator (ICOS) and ICOS-ligand (ICOSL) (70, 102), a co-stimulatory receptor/ligand pair known for its function for survival, proliferation, and cytokine secretion of T_H_ cell subsets (103). Moreover, ILC2s can process Ags and present peptides on MHC II. They express the co-stimulatory molecules, CD80 and CD86, and induce proliferation of T_H_2 cells, albeit to a lesser extent than professional APCs (92, 93). Interestingly, the expression of MHC II is higher on LN-, spleen-, and Peyer’s Patch (PP)-derived ILC2s than on peritoneal lavage-, bronchoalveolar lavage-, and lung-derived ILC2s. Therefore, lymphoid tissue-specific factors might be responsible for sustained MHC II expression.

**Figure 1:**
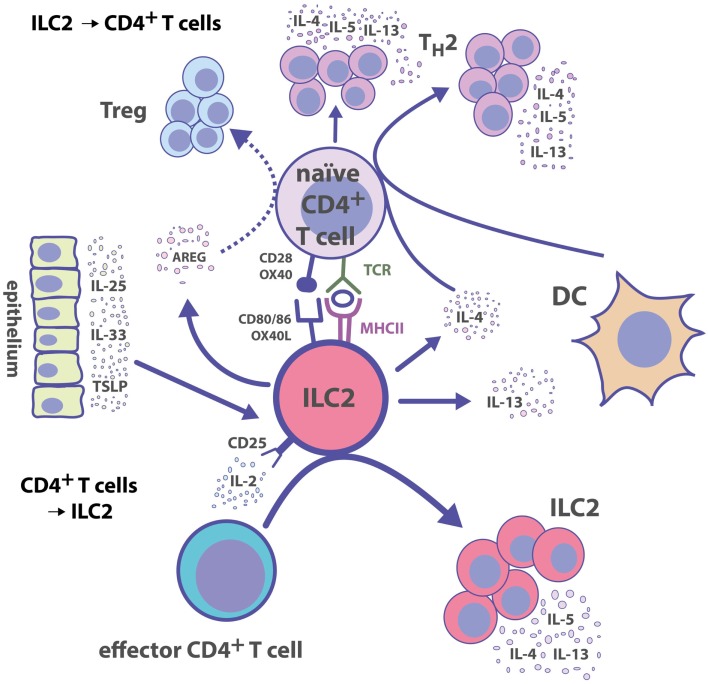
**Group 2 ILC–CD4^+^ T cell interactions**. ILC2s polarize CD4^+^ T cell responses toward T_H_2 immunity directly by presenting cognate Ag and by secreting T_H_2-inducing cytokines. Reciprocally, activated CD4^+^ T cells produce IL-2, which serves as a growth factor leading to the expansion of ILC2s.

Together with the finding that ILC2s can express MHC II and co-stimulatory molecules, the direct ILC2–T cell interaction not only promotes T_H_ responses but also extends to cytokine-mediated help from activated T_H_ cells for ILC2 effector functions. During the acute phase of *N. brasiliensis* infection, Rag2-deficient (*Rag2*^−^*^/^*^−^) mice show a similar expansion of ILC2s as *WT* mice. However, adaptive immune cells are required for prolonged ILC2 expansion and complete clearance of the infection (70). In a papain-induced inflammation model, IL-9 production by ILC2s is severely reduced in *Rag2*^−^*^/^*^−^ mice suggesting that cytokine secretion by ILC2s is also dependent on the adaptive immune system (68). *In vitro* co-culture of CD4^+^ T cells and ILC2s results in the upregulation of IL-4 mRNA in ILC2s, suggesting that T_H_ cells induce type 2 cytokine production by ILC2s (94). Additionally, activated CD4^+^ T cells in co-culture with ILC2s can directly induce ILC2 proliferation and IL-5/IL-13 secretion (92). This effect is partially impaired by adding anti-IL-2-neutralizing Abs but not by separating CD4^+^ T cells from ILC2s in transwell assays, suggesting an IL-2-driven feedback mechanism from activated CD4^+^ T cells to ILC2s (92). In line with this, treatment of mice with IL-2/anti-IL-2 complexes results in increased *in vivo* proliferation of ILC2s (62) and expansion of ILC2 progenitors in the bone marrow (BM) (45). IL-2 can also promote IL-9 release by ILC2s, whereas IL-33 induces the upregulation of the IL-2-receptor subunit CD25 on ILC2s (104). The induction of CD25 expression may help ILC2s to become more sensitive to T cell-derived IL-2. It is currently unclear to what extent ILC2s and T_reg_ cells, which express high levels of CD25, or other T_H_ subsets, compete for IL-2. Hence, the expression of CD25 by ILC2s may also reduce the availability of IL-2 for T cells. Based on these observations, we propose the following model (Figure 1): ILC2s can be rapidly activated by various alarm signals leading to the release of T_H_2-type cytokines, which help to induce T_H_2 cell responses and DC migration into LNs toward T cell zones. Further, activated ILC2s secrete AREG, and it remains to be investigated whether this can trigger T_reg_ cell responses. The cognate interaction between ILC2s and CD4^+^ T cells via MHC II–Ag presentation, co-stimulatory signals, and cytokines helps to amplify both ILC2 and CD4^+^ T cell responses.

## Group 3 ILCs

All ILC3 subsets depend on the transcription factor RORγt for their development (105–107), and produce the T_H_17-type cytokine IL-22 (107–111). IL-22 has a major role in protecting intestinal epithelial cells from bacterial infections and in promoting tissue repair through induction of epithelial cell proliferation and production of antimicrobial peptides (112). Group 3 ILCs can be phenotypically classified into a subset of fetal RORγt^+^ CD127^+^ CD117^+^ LTi cells (106, 113–116), and adult NCR^+^ or NCR^−^RORγt^+^ ILC3s (107, 108, 111, 117).

## Group 3 ILC–T Cell Interactions

**Figure 2:**
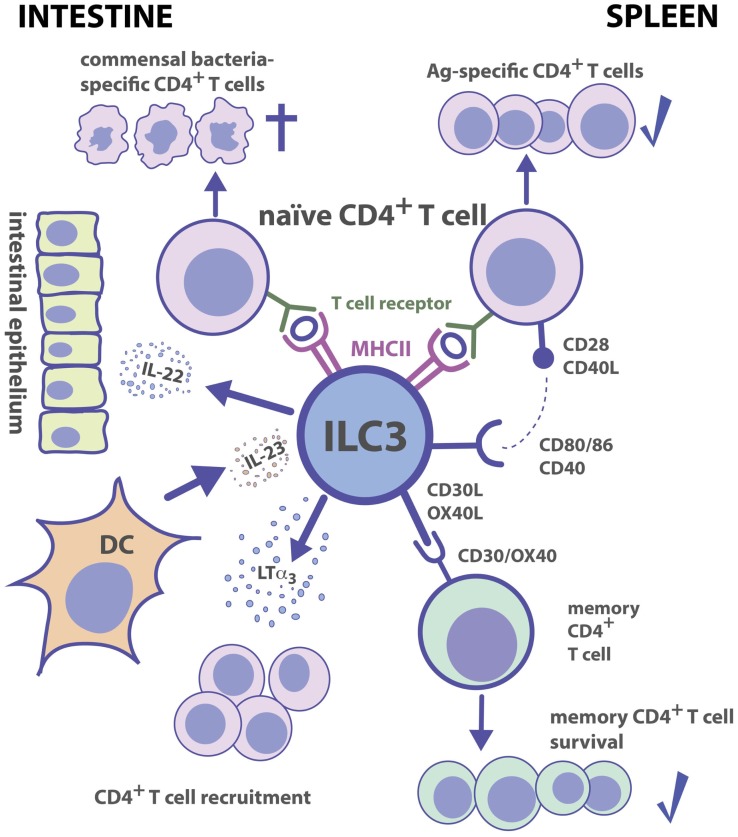
**Group 3 ILC–CD4^+^ T cell interactions**. Tissue localization greatly affects the outcome of Ag-dependent T cell–ILC3 interaction. Intestinal ILC3s maintain tolerance toward commensal microbiota, while splenic ILC3s are efficient in the induction of Ag-specific CD4^+^ T cell responses and memory CD4^+^ T cell survival.

ILC3s can modulate T_H_ cell immune responses in several ways. One pathway involves the development of lymphoid tissue and T cell zone stroma. Already before birth, the cellular crosstalk of fetal lymphotoxin (LT)α_1_β_2_-expressing LTi cells with mesenchymal stromal cells (MSCs) plays a pivotal role in the formation of LNs and PPs, in which immune responses are generated. Adult ILC3s retain the capacity to induce lymphoid tissue formation (118, 119). Following lymphocytic choriomeningitis virus (LCMV) infection in mice, the crosstalk between LTα_1_β_2_-expressing ILC3s and T cell zone fibroblastic reticular cells helps to restore the disrupted T-zone compartment and hence the structure to generate proper immune responses (120). Similarly, LTα_1_β_2_^+^ ILC3s can restore lymphoid follicle organization in the colon of mice infected with *Citrobacter rodentium* (121). The interaction of ILC3s with MSCs is also reciprocal. In humans, the crosstalk between LTα_1_β_2_^+^ ILC3s and marginal reticular cells (MRCs), a subset of marginal zone stromal cells, induces the production of MRC-derived survival factors for ILC3s, such as IL-7 (122). A second pathway, by which ILC3s can modulate T_H_ cell immune responses, is through altering the recruitment of CD4^+^ T_H_ cells. ILC3s are able to release soluble LTα_3_, which promotes the homing of CD4^+^ T_H_ cells to the gut lamina propria where they differentiate into functional T_H_ cell subsets (Figure 2) (123). In a model of airway inflammation, ILC3-derived IL-22 reduces CCL17 production by epithelial cells thereby limiting T_H_2 cell recruitment and immune responses to allergens in the lung (124). These data show that ILC3s have an impact on generating functional T cell compartments and recruitment of CD4^+^ T_H_ cells to mucosal sites.

In the adult spleen, ILC3s are localized in the marginal zone and around the central arterioles, and in LNs in proximity to high endothelial venules and interfollicular areas (122, 125–127). Because of the close association of splenic ILC3s to Ag-entry sites and T cells as well as their expression of the co-stimulatory molecules, CD30-ligand (CD30L) and OX40L, it has been assumed that they may directly interact with T cells during adaptive immune responses (125). Mice with a deficiency in CD30 and OX40 (*CD30*^−^*^/^*^−^*OX40*^−^*^/^*^−^ mice) lack proper memory Ab responses due to a failure in survival of primed CD4^+^ T_H_ cells (128). *In vitro*, ILC3s can promote survival of memory CD4^+^ T_H_ cells from *WT* but not from *CD30*^−^*^/^*^−^*OX40*^−^*^/^*^−^ mice suggesting that both CD30L and OX40L molecules expressed by ILC3s are essential for CD4^+^ T_H_ memory responses (128). This possibility was supported by an *in vivo* study, which identified ILC3s as the key players in the maintenance of CD4^+^ memory T_H_ cells (Figure 2) (129).

A third mechanism by which ILC3s interact with CD4^+^ T_H_ cells is through receptors required for immune recognition. ILC3s isolated from various tissues of fetal, neonatal, and adult mice express MHC II and MHC II-associated gene transcripts (44, 113, 130–132). NCR^−^ILC3s are able to internalize, process, and present foreign Ags to CD4^+^ T_H_ cells (130, 131). Under non-inflammatory conditions, ILC3s express neither CD40 and CD80 nor CD86 (130, 131). However, following stimulation with IL-1β splenic but not intestinal, NCR^−^ILC3s can upregulate co-stimulatory molecules (130). A recent study confirmed that even after toll-like receptor ligand (TLRL) or pro-inflammatory cytokine exposure, intestinal ILC3s do not upregulate co-stimulatory molecules (133). The finding that mLN-derived ILC3s are as well unable to express co-stimulatory molecules upon stimulation is likely due to the fact that ILC3s found in the mLNs are originally intestinal ILC3s, which were trafficking from the intestine to the mLNs (127). It is noteworthy that genome-wide transcriptional profiling of splenic ILC3s reveals an enrichment for genes involved in cell activation and immune responses (63). In contrast to splenic ILC3s, intestinal ILC3s express the activation marker, CD69 (44), a glycoprotein involved in establishing oral tolerance (134) and limiting dextran sodium sulfate (DSS)-induced inflammation (135). Moreover, ILC3s present in the small intestine express neuropilin-1 (Nrp1) (44), which promotes T_reg_ cell survival and functional activity (136–138). It is therefore conceivable that ILC3s exert tissue-specific immune functions with immunogenic versus tolerogenic activity in the spleen and intestine, respectively. This hypothesis is further supported by the notion that splenic NCR^−^ILC3s promote CD4^+^ T_H_ cell responses *in vitro* and *in vivo*, whereas intestinal ILC3s fail to efficiently stimulate CD4^+^ T_H_ cells (Figure 2) (130). In mice, intestinal ILC3s express lower levels of MHC II as compared to ILC3s identified in other tissues (130, 131, 133). Together with the observation that intestinal ILC3s lack co-stimulatory molecules, this may contribute to maintaining intestinal T cell tolerance, similar to immature DCs expressing low surface levels of MHC II and co-stimulatory molecules (139).

Hepworth et al. reported the development of spontaneous intestinal inflammation in mice lacking MHC II exclusively on ILC3s (ILC3Δ^MHCII^ mice) and found a role for intestinal ILC3s in limiting commensal bacteria-specific pro-inflammatory colonic CD4^+^ T_H_ cell responses through induction of PD (131, 133). Since other laboratories failed to detect spontaneous signs of inflammation in ILC3Δ^MHCII^ mice (130, 132), it is possible that the development of immunopathology is triggered by microbial co-factors. In the intestine, ILC3s can inhibit T_H_17 cell-mediated inflammation through AHR signaling, release of IL-22, and by preventing the expansion of aberrant segmented filamentous bacteria (SFB) (140). In pediatric Crohn’s disease (CD) patients, MHC II levels on intestinal ILC3s are significantly reduced, and such low expression correlates with increased frequencies of colonic T_H_17 cells and circulating commensal bacteria-specific IgG (133). This study is the first to describe an association of ILC3-mediated Ag presentation and control of commensal bacteria-specific adaptive immunity in humans. It remains unclear which are the mechanisms that underlie loss of MHC II in CD patients and whether this is sufficient to trigger inflammatory bowel disease. Together, these findings suggest that intestinal ILC3s can inhibit expansion of T_H_17 cells and immunopathology after exposure to pro-inflammatory stimuli.

Analogously to ILC2–T cell interactions, the crosstalk between ILC3s and CD4^+^ T_H_ cells might be bidirectional and depends on cytokines. This is further supported by the findings that the presence of the adaptive immune system has an effect on the number and IL-22 production of intestinal ILC3s, most likely through competition for growth factors (141, 142). Human and activated mouse ILC3s produce IL-2 (19, 130), and conversely, TLR2-driven proliferation of human ILC3s is partially dependent on IL-2 (19). Availability of IL-2 alone or in combination with Pam3Cys promotes increased CD25 expression in human ILC3s suggesting that CD25 expression might help ILC3s to win the competition for IL-2 against T cells (19). Moreover, there is some evidence that mouse ILC3s have a higher capacity to bind IL-2 than activated CD4^+^ T_H_ cells (133). Therefore, the availability of IL-2 can restrict ILC3 and T_H_ responses as a result of receptor density, efficiency of binding, and kinetics of IL-2 consumption.

## Immune Homeostasis in the Gut: Tolerance Versus Inflammation

The critical question regarding maintenance of immune homeostasis is where, when, and how immune responses prevent tissue injury. The intestine is a prime example that has been extensively studied with respect to cellular networks and pathways patrolling tissue integrity and regulating inflammation. T_reg_ and T_H_17 cells are the most abundant CD4^+^ T_H_ cells in the intestinal mucosa under steady state (143–145). The balance between the two subsets is crucial for the outcome of mucosal immune responses (146). Commensal bacteria have a specific impact on the number of both T_H_ subsets (147) and on the capacity of ILC3s to regulate T_H_ subset responses (148). On the other hand, ILC3s contribute to maintenance of intestinal epithelial barrier function thereby limiting microbes entry and inflammatory T_H_ cell responses (108, 109, 117, 141, 148). Whereas under steady-state conditions, intestinal ILC3s produce high levels of IL-22, the production of IL-17 is rather low (44). Importantly, T_H_17 cells are induced by SFB (149, 150) by a mechanism that requires SFB presentation by DCs (132, 151). In contrast, ILC3 presentation of Ag prevents amplification of SFB-independent T_H_17 cells (132). In line with this, the expansion of SFB and pathogenic T_H_17 cells inversely correlates with the number of intestinal ILC3s (140). In an IL-17-dependent autoimmune mouse model, it was recently shown that SFB colonization was associated with enhanced auto-Ab titers (152). The increase in IL-17-producing cells, as observed in CD patients (153), is probably not sufficient *per se* to induce immunopathology. Specificity of inflammatory T_H_ cells, intestinal infections, pro-inflammatory bystander cells, and loss of functional T_reg_ cells might be required to trigger intestinal inflammation.

All these studies published in recent years raised the question of whether and how ILC–T cell interactions regulate pro- or anti-inflammatory responses in the gut. Since ILC3s can prevent dissemination of commensal bacteria in the gut and commensal bacteria-specific T_H_ cell responses (123, 131, 132, 148), they probably promote an immunological tolerogenic state in the gut. In addition, the production of GM-CSF by ILC3s has the potential to enhance iT_reg_ cell numbers and function thereby promoting intestinal homeostasis (154). In some colitis models, however, ILC3s were reported to enhance intestinal inflammation (13, 15), and pathogenic ILC1 numbers were increased in patients with CD (16, 41). The functional polarization toward IFNγ-producing ILC1s or IL-22-producing ILC3s appears to depend on tissue-specific and pro-inflammatory conditions. Environmental changes may immediately affect the ratio and/or polarization of ILC and T cell subsets. For example, induction of pro-inflammatory cytokines, such as IL-23, was shown to counteract the responsiveness toward IL-33, and the generation of iTregs in the intestine (98). As for T_H_ cell differentiation, it is likely that the amount of cytokines determines ILC cytokine polarization. Under homeostatic conditions, the intestine provides a microenvironment enriched of cytokines with inhibitory effects, such as TGF-β. At high dose, TGF-β inhibits T_H_17 responses, whereas low-dose TGF-β promotes T_H_17-differentiation (155–157). A similar impact of cytokine concentrations for immune homeostasis has also been discussed for IL-22 (158). Therefore, excessive release of cytokines by ILCs may contribute to immunopathology, whereas under steady-state conditions, ILCs rather promote epithelial tissue integrity and tolerogenic T cell responses. During inflammation, ILC3s can switch off RORγt expression, which may eventually be regained at later time points. The modulation of cytokine receptors during a critical time window of ILC activation and ILC-T cell interaction might also contribute to prevent excessive immunopathology. This has been shown for a number of receptors controlling growth and survival of both ILCs and T cells. Finally, the polarization toward protective versus inflammatory response in the gut likely requires a tight balance between temporal regulation, amount, and combination of cytokines co-expressed by individual ILCs.

## Conclusion

Our understanding of immune homeostasis has been challenged by the notion that environmental factors, including commensal bacteria and nutritional components, as well as cholinergic and metabolic signals can regulate immune functions and pro-inflammatory processes. ILCs are important “early sentinel” cells, which connect innate and adaptive immunity by sensing environmental changes, such as infections and inflammation and by the release of immuno-regulatory cytokines. They not only contribute to T cell immune homeostasis by promoting T_H_ cell differentiation and effector functions but can also directly interact with CD4^+^ T_H_ cells. Both ILC2s and ILC3s internalize and present Ag to T_H_ cells. Considering the fact that the number of ILCs in most tissues is rather low as compared to other immune cells, they appear to have a surprising *in vivo* impact on immune homeostasis. The localization of ILCs in relatively high density at Ag-entry sites and T cell areas as well as bystander effects on classical DCs might explain this effect. In addition, advances in two-photon microscopy have shown that several CD4^+^ T_H_ cells are often clustering with the same APC, a fact that may increase local cytokine concentrations for optimal cell–cell interactions. The capacity to elicit cognate T_H_ cell proliferation or rather prevent T_H_ cell responses strongly depends on environmental factors and the nature of Ag, and it will be important to further investigate the mechanisms by which ILCs prevent or promote T cell responses in various tissues. For example, it will be interesting to unravel whether ILCs can express inhibitory receptors and/or collaborate with T_reg_ cells. Finally, there are clearly cytokine-driven reciprocal effects between ILCs and T cells, which might help to coordinate and/or limit immune responses. Taken together, a better understanding of the regulation of cytokine expression by ILCs and their interaction with T cells will help to develop new strategies to treat inflammatory diseases in humans.

## Conflict of Interest Statement

The authors declare that the research was conducted in the absence of any commercial or financial relationships that could be construed as a potential conflict of interest.
